# Arrhythmogenic Cardiomyopathy and Skeletal Muscle Dystrophies: Shared Histopathological Features and Pathogenic Mechanisms

**DOI:** 10.3389/fphys.2020.00834

**Published:** 2020-07-30

**Authors:** Shanshan Gao, Suet Nee Chen, Carlo Di Nardo, Raffaella Lombardi

**Affiliations:** ^1^Division of Cardiology, Department of Medicine, University of Colorado, Aurora, CO, United States; ^2^Division of Cardiology, Department of Advanced Biomedical Sciences, Federico II University of Naples, Naples, Italy

**Keywords:** arrhythmogenic cardiomyopathy, skeletal muscle dystrophies, fibroadiposis, inflammation, molecular pathogenesis

## Abstract

Arrhythmogenic cardiomyopathy (ACM) is a heritable cardiac disease characterized by fibrotic or fibrofatty myocardial replacement, associated with an increased risk of ventricular arrhythmias and sudden cardiac death. Originally described as a disease of the right ventricle, ACM is currently recognized as a biventricular entity, due to the increasing numbers of reports of predominant left ventricular or biventricular involvement. Research over the last 20 years has significantly advanced our knowledge of the etiology and pathogenesis of ACM. Several etiopathogenetic theories have been proposed; among them, the most attractive one is the dystrophic theory, based on the observation of similar histopathological features between ACM and skeletal muscle dystrophies (SMDs), such as progressive muscular degeneration, inflammation, and tissue replacement by fatty and fibrous tissue. This review will describe the pathophysiological and molecular similarities shared by ACM with SMDs.

## Introduction

Arrhythmogenic cardiomyopathy (ACM) is a primary heritable disease of the myocardium, clinically characterized by increased risk of ventricular arrhythmias and sudden cardiac death (SCD) (Towbin et al., 2019).

ACM includes arrhythmogenic right ventricular cardiomyopathy (ARVC), which affects the right ventricle (RV); left dominant arrhythmogenic cardiomyopathy (LD-ACM), in which the left ventricle is the first chamber to be affected; and biventricular ACM (Sen-Chowdhry et al., 2008; Miles et al., 2019).

The histological feature of ACM is the progressive replacement of the myocardium with fibrotic or fibrofatty tissue, typically starting from the epicardium (Lombardi and Marian, 2010; Basso et al., 2011).

Three main etiopathogenetic theories have been proposed to explain the origin and development of the ACM phenotype: (Basso et al., 1996; Towbin et al., 2019) the dysontogenetic (dysplasia) theory considers ACM as a developmental disorder of the RV (Marcus et al., 1982; Sen-Chowdhry et al., 2008), the myocarditis theory based on the evidence of inflammation in ACM hearts (Basso et al., 1996; Campian et al., 2010; Miles et al., 2019; Protonotarios et al., 2019) and the dystrophic theory, based on the histopathological similarities between ACM and skeletal muscle dystrophies (SMDs) (Pearce et al., 1981; Hadar et al., 1983; Lombardi and Marian, 2010; Basso et al., 2011). According to the dysontogenetic theory, the disease was interpreted as a congenital defect of the development of the right ventricular myocardium and hence called “right ventricular dysplasia” (Marcus et al., 1982). Later on, evidence from genetic, morphological, and clinical studies showed that ACM was not a structural defect present at birth but a genetic progressive disease of the myocardium, associated with a high risk of life-threatening arrhythmias (Basso et al., 1996, 2010, 2011). For this reason, the term dysplasia was replaced, and the condition became known as “arrhythmogenic RV cardiomyopathy,” and the disease was listed, together with hypertrophic, restrictive, and dilated variants, in the WHO classification of cardiomyopathies in 1995 (Richardson et al., 1996).

Although ACM is a cardiac pathology while SMD affects mainly the skeletal muscle, the two diseases share histological features as well as molecular and cellular pathogenic mechanisms.

ACM and SMD are both genetically transmitted and show similar histopathological hallmarks, namely muscle degeneration, inflammation, and tissue replacement by fibrosis and fat.

Myocardium loss in ACM is the consequence of myocyte death by apoptosis and/or necrosis, which is accompanied by inflammation followed by abnormal fibrofatty repair (Basso et al., 1996, 2011; Pilichou et al., 2009). Similarly, muscle degeneration, inflammation, fat infiltration, and fibrosis have been detected in the muscles of patients and mouse models of SMD, mainly in Duchenne muscle dystrophy (DMD) (Pearce et al., 1981; Hadar et al., 1983; Nowak and Davies, 2004; Consalvi et al., 2013).

Inflammation is a key feature of both diseases and precedes the fibrofatty infiltration (Basso et al., 1996; Lombardi and Marian, 2010). Nevertheless, whether inflammation plays a primary role or is a secondary response to cell death remains elusive.

Although the primary presenting symptom in SMDs is skeletal muscle weakness, cardiac muscle may also be similarly affected. Indeed, cardiomyopathies are an increasingly recognized manifestation of SMDs and contribute significantly to the morbidity and mortality (Kamdar and Garry, 2016).

DMD is the most common form of SMD. DMD is inherited in a X-linked recessive manner and is caused by out-of-frame mutations, which result in the absence of functional dystrophin protein. Becker muscular dystrophy (BMD) is another X-linked muscular dystrophy showing a milder clinical course than DMD. BMD is also caused by mutations in the *DMD* gene, but mutations in BMD tend to be in-frame and result in abnormal and less functional dystrophin instead than in the complete absence of the protein (Monaco et al., 1988; Kamdar and Garry, 2016). Research over the last 20 years has significantly advanced our knowledge of the etiology and pathogenesis of both ACM and SMD. Two main pathways, the Wnt signaling and the Hippo pathway, are affected in both diseases (Garcia-Gras et al., 2006; Lombardi et al., 2011; Chen et al., 2014). Furthermore, the heart and skeletal muscle contain a subset of analogous resident mesenchymal progenitor cells, identified by the surface marker platelet-derived growth factor receptor alpha (PDGFRA) and called fibroadipocyte progenitors, which, in the presence of chronic myocyte injury induced by the causal mutation, differentiate to adipocytes and fibroblasts (Uezumi et al., 2010, 2011; Gurha et al., 2016; Lombardi et al., 2016; Malecova et al., 2018).

This review describes similarities in genetics, histology/imaging features, and pathogenic mechanisms between ACM and SMD.

### Genetics

Despite that the familial background of ACM was known from late 1980s (Nava et al., 1988, 2000), the first causal mutation was identified 20 years later in a rare recessive syndrome known as Naxos disease, characterized by typical ARVC associated with wooly hair and palmoplantar keratoderma (McKoy et al., 2000). The mutation was a 2 bp deletion in the gene encoding for plakoglobin (*JUP*) (McKoy et al., 2000), a protein of the desmosomes and adherens junctions, which are part of a more complex functional unit, responsible for structural integrity and synchronized contraction of the cardiac tissue, named intercalated disk (ID). IDs include, in addition to desmosomes, several other specialized structures, mainly fascia adherens and gap junctions (Sheikh et al., 2009; Swope et al., 2013). Desmosomes are complex structures not only responsible for cell-cell attachment but also regulators of signaling pathways (Swope et al., 2013).

After the discovery of *JUP* gene, additional causal autosomal dominant mutations were identified in desmosome genes, such as plakophilin-2 (*PKP2*) (Gerull et al., 2004; den Haan et al., 2009), desmoplakin (*DSP*) (Rampazzo et al., 2002), desmoglein-2 (*DSG2*) (Pilichou et al., 2006; Gehmlich et al., 2010), and desmocollin-2 (*DSC2*) (Syrris et al., 2006); in addition, ACM autosomal dominant forms due to *JUP* (De Deyne et al., 2006) and recessive forms due to *DSP* (Norgett et al., 2000) and *DSC2* (Al-Sabeq et al., 2014) mutations have been identified, usually in the context of cardiocutaneous syndromes. Mutations in desmosomal genes are identified in approximately two-thirds of the affected probands; hence ACM is commonly considered a disease of the desmosomes.

ACM mutations have also been identified in non-desmosomal genes encoding for adherens junction components such as catenin-α3 and cadherin 2 (van Hengel et al., 2013; Mayosi et al., 2017; Turkowski et al., 2017); nuclear lamina proteins lamin A/C (*LMNA*) (Quarta et al., 2012) and transmembrane protein 43 (*TMEM43*) (Merner et al., 2008); cytoskeletal proteins desmin (*DES*) (van Tintelen et al., 2009; Klauke et al., 2010; Bermudez-Jimenez et al., 2018) and filamin C (*FLNC*) (Ortiz-Genga et al., 2016; Hall et al., 2019; Brun et al., 2020); the sarcomere protein titin (*TTN*) (Taylor et al., 2011); ion channels such as phospholamban (*PLN*) (van der Zwaag et al., 2012, 2013; van der Heijden and Hassink, 2013), ryanodine receptor 2 (*RYR2*) (Tiso et al., 2001), and sodium voltage-gated channel alpha subunit 5 (SCN5A) (Te Riele et al., 2017); and transforming growth factor β3 (*TGFB3*) (Beffagna et al., 2005; Table 1).

**TABLE 1:** List of causal genes for ACM.

**Gene**	**Encoded protein**	**Estimated frequency (%)**	**Features**	**Mode of inheritance**	**References**
**Desmosome**					
*PKP2*	Plakophilin 2	∼40	Haploinsufficiency; ACM	AD, AR	Nava et al., 1988, 2000
*DSP*	Desmoplakin	∼16	Associated with LV-dominant disease; Carvajal syndrome	AD, AR	McKoy et al., 2000; Rampazzo et al., 2002
*DSG2*	Desmoglein 2	∼10	Overlap with DCM	AD	Sheikh et al., 2009; Swope et al., 2013
*DSC2*	Desmocollin 2	∼8	ACM	AD, AR	Gerull et al., 2004; Pilichou et al., 2006
*JUP*	Junction plakoglobin	Rare	Naxos disease; autosomal dominant ACM	AD, AR	den Haan et al., 2009; Joe et al., 2010
**Adherens junction**					
*CTNNA3*	Catenin-α3	Rare	Incomplete penetrance; normal plakoglobin localization	AD	Gehmlich et al., 2010
*CDH2*	Cadherin 2	Rare	No specific genotype–phenotype relationship identified	AD	De Deyne et al., 2006; Syrris et al., 2006
**Nucleus/Cytoskeleton**					
*LMNA*	Lamin A/C	Rare	DCM phenotype, conduction defects, arrhythmias and high risk of sudden cardiac death; muscle dystrophies; lipodystrophies; progeria	–	Norgett et al., 2000; Te Riele et al., 2017
*TMEM43*	Transmembrane protein 43	Rare	Fully penetrant; affected men more severely than women; LV involvement; muscle dystrophy	AD	Al-Sabeq et al., 2014
*DES*	Desmin	Rare	Fully penetrant; associated with LV and RV-dominant ACM, DCM and skeletal myopathies	AD	Tiso et al., 2001; van Hengel et al., 2013; van der Zwaag et al., 2013; van der Heijden and Hassink, 2013; Mayosi et al., 2017; Turkowski et al., 2017
*FLNC*	Filamin C	Rare	Associated with Left-dominant ACM, high risk of arrhythmias and sudden death	–	Merner et al., 2008; Klauke et al., 2010; Quarta et al., 2012
**Sarcomere**					
*TTN*	Titin	Rare	Higher risk of supraventricular tachycardia and progression to heart failure; tibial muscular dystrophy	–	van Tintelen et al., 2009
**Ion transport**					
*PLN*	Phospholamban	Rare	Low prevalent and cause DCM and ACM	–	Ortiz-Genga et al., 2016; Bermudez-Jimenez et al., 2018; Hall et al., 2019
*RYR2*	Ryanodine receptor 2	Rare	Overlap with DCM	AD	Brun et al., 2020
*SCN5A*	Nav1.5	Rare	Prolonged QRS interval	–	Taylor et al., 2011
**Cytokines**					
*TGFB3*	Transforming growth factor-β3	Rare	No specific genotype–phenotype relationship identified	–	van der Zwaag et al., 2012

**AD, autosomal dominant; AR, autosomal recessive; ACM, Arrhythmogenic cardiomyopathy; DCM, Dilatative Cardiomyopathy; LV, left ventricle; RV, right ventricle; SCN5A, Sodium Voltage-Gated Channel Alpha Subunit 5; Nav1.5, α-subunit of the cardiac sodium channel complex.**

Interestingly, *DES* (Melberg et al., 1999; van Spaendonck-Zwarts et al., 2011; Hedberg et al., 2012), *LMNA* (Capell and Collins, 2006; Quarta et al., 2012), *TMEM43* (Merner et al., 2008; Liang et al., 2011; Mukai et al., 2019), and *TTN* (Hackman et al., 2002, 2008; Pollazzon et al., 2010; Taylor et al., 2011; Misaka et al., 2019) have been associated with either cardiomyopathies or SMD. Hence, patients with primary myopathy due to a mutation in one of these genes should be screened for cardiac involvement.

Desmin is the main intermediate filament and is highly expressed in both skeletal and cardiac muscle cells; therefore, *DES* mutations frequently cause concomitant skeletal-muscle and cardiac phenotype (van Spaendonck-Zwarts et al., 2011).

Mutations in the *LMNA* gene, encoding lamin A and lamin C, cause a wide range of diseases: Emery-Dreifuss muscular dystrophy (EDMD) types 2 and 3, limb girdle muscular dystrophy (LGMD) type 1B, cardiomyopathy, lipodystrophies, peripheral neuropathies, and progeria syndromes. These diseases are collectively known as laminopathies (Capell and Collins, 2006). Conduction disorders, atrial fibrillation, ventricular tachycardia, and increased risk of sudden cardiac death are typical features of LMNA-associated cardiomyopathies. Mutations in *TMEM43* may cause ACM and EDMD (Merner et al., 2008; Liang et al., 2011; Mukai et al., 2019). It is not clear how defects in these nuclear membrane proteins result in the phenotype development; possibly, the mutant proteins may increase mechanical stress to the nucleus or alter gene expression through interaction with the chromatin (Chen et al., 2019; Cheedipudi et al., 2019).

*TTN* mutations cause different skeletal phenotypes (such as tibial muscular dystrophy, LGMD type 2J, EDMD, hereditary myopathy with early respiratory failure, central core myopathy, centronuclear myopathy), collectively known as titinopathies. The severity of the myopathy and the cardiac involvement in titinopathies are determined by the position and type of *TTN* mutation (Hackman et al., 2008; Misaka et al., 2019). Moreover, some *TTN* mutations cause isolated cardiomyopathy, mainly DCM but also HCM or ACM, without skeletal muscle involvement (Taylor et al., 2011; Misaka et al., 2019).

DMD and BMD are caused by mutations in the gene encoding dystrophin, a rod-shaped cytoplasmic protein, which is part of the dystroglycan complex (DGC), the multimeric complex that forms a structural link between the filamentous (F)-actin cytoskeleton and the extracellular matrix (ECM) in both cardiac and skeletal muscle and provides mechanical support to the skeletal or cardiac plasma membrane during contraction (Ervasti and Campbell, 1993; Rybakova et al., 2000). It has been shown that the specific dystrophin mutations affect the incidence and severity of cardiomyopathy and response to treatment (Jefferies et al., 2005).

### Histological Features and Inflammation

**FIGURE 1:**
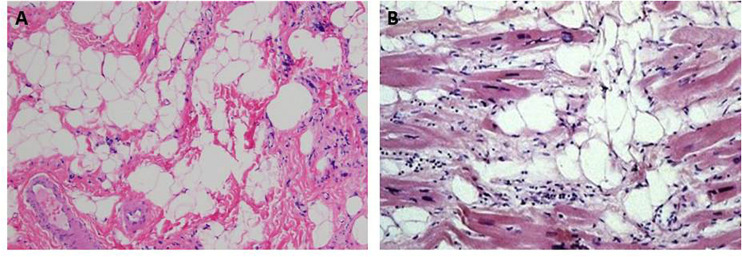
Hematoxylin & eosin images showing fibroadiposis in human skeletal muscle dystrophies (SMD) and in arrhythmogenic cardiomyopathy (ACM). **(A)** The pathological aspect of the skeletal muscle observed in Duchenne muscle dystrophy. **(B)** shows the typical histological aspect of the heart tissue in ACM. Panel (A) from Hiser (2020). Panel (B) from Thiene et al. (2007).

ACM and SMD share similar histopathological features consisting of progressive muscular degeneration due to apoptosis or necrosis, inflammatory infiltrates, and fibrofatty tissue replacement (Pearce et al., 1981; Hadar et al., 1983; Basso et al., 1996, 2011; Mallat et al., 1996; Corrado et al., 1997; Nowak and Davies, 2004; Pilichou et al., 2009; Consalvi et al., 2013; Figure 1). Interestingly, fibrosis in ACM is present not only in the form of fibrofatty infiltration but also as interstitial fibrosis, with or without adiposis (Basso et al., 2008; Marcus et al., 2010).

Inflammation is a key feature of both ACM and SMD and is usually detected at the early stages of the disease before the development of fibroadiposis.

Infiltration of inflammatory cells in the heart has been found in over 70% of ARVC patients and in ACM mouse models (Thiene et al., 1991; Basso et al., 1996, 2011; Corrado et al., 1997; Pilichou et al., 2009; Li et al., 2011; Elliott et al., 2019): patchy mononuclear inflammatory infiltrates of CD3 + T lymphocytes (Elliott et al., 2019) and CD45 +; CD68 + (Basso et al., 1996; Elliott et al., 2019) macrophages are observed in association with dying myocytes, suggesting that the pathological process may be immunologically mediated (Thiene et al., 1991; Basso et al., 1996; Corrado et al., 1997; Elliott et al., 2019).

Similarly, the presence of macrophages, CD4 + and CD8 + T cells, natural killer T cells, neutrophils, and eosinophils has been described in the skeletal muscle of patients with DMD and of mdx mice, a widely used animal model of DMD (Arahata and Engel, 1984; Spencer and Tidball, 2001; Spencer et al., 2001; Vetrone et al., 2009; Lozanoska-Ochser et al., 2018). A recent study has shown that T lymphocytes infiltrate mdx mouse dystrophic muscles prior to the occurrence of necrosis, suggesting a primary role of this cell type in the onset of the disease. Furthermore, the same study demonstrated that inhibition of the protein kinase C θ, a key regulator of T-cell activation, markedly diminishes the size of the inflammatory cell infiltrates and reduces muscle damage (Lozanoska-Ochser et al., 2018).

Inflammation in ARVC patients has also been confirmed by detection of increased plasma levels of interleukin (IL)-1β, IL-6, and tumor necrosis factor alpha (TNF-α) (Campian et al., 2010). Similarly, DMD patients and mdx mice present with higher serum levels of inflammatory cytokines as compared with healthy subjects (Barros Maranhao et al., 2015; Cruz-Guzman Odel et al., 2015; Pelosi et al., 2015).

The empirical observation that immunosuppressive drugs, such as glucocorticoids, can improve muscle strength in patients and in animal models of DMD furthers supports a role for the immune system in the pathogenesis of the disease (Wehling-Henricks et al., 2004; Hussein et al., 2006).

Although the presence of inflammation is widely recognized in both ACM and SMD, its origin and role as a primary event or secondary response to myocyte damage is unknown.

The role of autoimmunity as a trigger for inflammation is a new research field in both ACM and SMD. However, so far, the evidence supporting the involvement of autoimmune response in the pathogenesis of these diseases is still limited, and further studies are needed for the identification of specific molecular and cellular players.

Although the infiltration of immune cells in dystrophic muscle is viewed as a generalized inflammatory response (Arahata and Engel, 1984; Spencer and Tidball, 2001; Spencer et al., 2001; Vetrone et al., 2009), several studies suggest that an autoimmune T-cell-mediated response to specific ill-muscle antigens may also be involved in the pathogenesis of SMDs (Gussoni et al., 1994; Vetrone et al., 2009; Burzyn et al., 2013; Villalta et al., 2015).

Recent deep-sequencing studies examining the T-cell receptor (TCR) repertoire of regulatory T cells (Tregs) in mdx muscle revealed an enrichment of several TCR rearrangements (Burzyn et al., 2013), suggesting that Tregs react to multiple self-antigens in dystrophic muscle. Thus, patients may retain a pool of dystrophin-reactive T cells, which may be further activated by expression of mutant dystrophin or dystrophin introduced exogenously by gene therapy. Indeed, the presence of an autoimmune response in muscle dystrophies was described in a clinical gene therapy trial in 2010 in which circulating antidystrophin T cells were unexpectedly detected in DMD patients before transgene delivery (Mendell et al., 2010). The autoimmune response was induced by epitopes contained in the truncated dystrophin encoded by the endogenous gene after spontaneous in-frame splicing (Mendell et al., 2010).

Another recent study has confirmed that a substantial number of DMD patients present with a pre-existing pool of circulating dystrophin-reactive T cells (Flanigan et al., 2013).

Likewise, the cause of the non-infectious myocarditis in ACM could plausibly be due to autoimmunity. Specific serum antidesmoglein-2 autoantibodies have been detected in ARVC patients regardless of the underlying mutation as compared with normal subjects and non-ARVC cardiomyopathies (Chatterjee et al., 2018). A recent study has shown the presence of serum antiheart autoantibodies (AHAs) and anti-intercalated disk autoantibodies (AIDAs) in the majority of familial and in almost half of sporadic ARVC cases, including some healthy relatives (Caforio et al., 2020). Furthermore, the serum levels of these autoantibodies in affected individuals were associated with disease severity (Caforio et al., 2020). The findings suggest a primary role of autoimmunity in the pathogenesis of ACM, as observed previously in primary dilated cardiomyopathy (Caforio et al., 1994), and open the stage to the potential therapeutic use of immunosuppression in biopsy-proven virus-negative autoantibody-positive inflammatory ACM.

The pathobiology responsible for an autoimmune response in ACM patients is still unknown; likewise, further studies are necessary to establish to what degree autoimmunity participates in the pathophysiology of the disease. One hypothesis is that the ACM causal mutation may induce the expression of a protein with unmasked “cryptic” epitopes; alternatively, myocyte damage may lead to the release of autoantigens that stimulate the immune system to generate autoantibodies.

Longitudinal studies will eventually clarify whether AHA and AIDA may be used as biomarkers to predict the development of the disease in healthy relatives.

### Non-invasive Tissue Characterization

Currently, the preferred imaging technique in both ACM and SMD is magnetic resonance imaging (MRI), which combines structural and functional evaluation with non-invasive tissue characterization (Parsai et al., 2012).

Tissue characterization by MRI is mainly based on the detection of changes in proton relaxation times T1 (also known as longitudinal relaxation) and T2 (also known as transverse relaxation). T1-weighted sequences are used to identify fat infiltration and diffuse fibrosis, while T2-weighted imaging is mainly used to detect the presence of edema. Moreover, T1-weighted imaging after infusion of gadolinium, also known as late gadolinium enhancement (LGE), is used to detect focal fibrosis. In cardiology, this technique is essential in the differential diagnosis of ischemic cardiomyopathy, in which LGE is localized in an area of coronary artery distribution and always involves the subendocardial region, versus non-ischemic cardiomyopathy, in which LGE does not occur in a specific coronary artery territory and is often midwall or epicardial rather than subendocardial or transmural (Parsai et al., 2012). Moreover, from the LGE distribution patterns, it is possible to make an etiological diagnosis (Kramer, 2015; Patel and Kramer, 2017).

Cardiac MRI has an important role in the clinical management of ACM because it allows the identification of minor tissue abnormalities, before the onset of morphofunctional abnormalities observed by echocardiography (Sen-Chowdhry et al., 2007, 2008; Sen-Chowdhry and McKenna, 2008). Furthermore, it has been shown that LGE distribution pattern differs among ACM subtypes and between carriers of desmosomal versus non-desmosomal pathogenic variants (Sen-Chowdhry et al., 2007; Sen-Chowdhry and McKenna, 2008; Segura-Rodriguez et al., 2019). Septal LGE is present in > 50% of cases of LD-ACM, unlike ARVC in which septal involvement is rare (Sen-Chowdhry et al., 2008). Furthermore, ACM patients with non-desmosomal variants usually show a circumferential subepicardial LV-LGE pattern, while those with desmosomal variants are more likely to have RV-LGE (Segura-Rodriguez et al., 2019).

Tissue characterization of skeletal muscle by MRI has an important role in disease staging and evaluation of therapeutic response for a number of neuromuscular diseases, including DMD (Finanger et al., 2012). T1-weighted imaging in the skeletal muscle of DMD patients shows higher T1 values at the early stages of the disease, which go down with increasing fatty replacement and clinical deterioration (Matsumura et al., 1988; Finanger et al., 2012). Moreover, it has been shown that the mean T2 relaxation time of thigh muscles in DMD correlates significantly with the mean fat fraction (Yin et al., 2019) and the severity of muscle weakness (Kim H.K. et al., 2013).

Gadolinium, which in normal muscle remains extracellular, is taken up into the muscle fibers with damaged membranes (Thibaud et al., 2007). Hence, because dystrophin deficiency renders muscle fibers susceptible to contraction-induced injury, a higher amount of gadolinium uptake has been found in the muscles of DMD patients after stepping exercise as compared with controls (Garrood et al., 2009).

It is known that patients with dystrophinopathies may develop dilated cardiomyopathy. Cardiac MRI provides accurate assessments of left ventricular size and function in patients with DMD and BMD (Hagenbuch et al., 2010). Cardiac MRI, with the use of strain technique, allows to identify cardiac involvement in the early stages, before the evidence of functional impairment at the echocardiography. The presence of LGE in the dystrophic heart is a sensitive early detection tool, as it usually precedes LV systolic dysfunction; moreover, the extent of fibrosis has been associated with an increased risk of progression toward ventricular dysfunction and increased mortality (Silva et al., 2007; Patel and Kramer, 2017). DMD and BMD patients with associated cardiomyopathy present a characteristic non-ischemic LGE pattern localized in the posterobasal region of the left ventricle, which starts from the subepicardium. Over the years, with the deterioration of the cardiac dysfunction, the LGE extends toward transmurality and to other myocardial segments (i.e., septum) (Puchalski et al., 2009); the late involvement of the septum differentiate the DMD cardiomyopathy from left-sided ACM, in which the septum is already affected in the early stages of the disease.

### Pathogenic Molecular Pathways

Mutations in desmosomal genes account for about two-thirds of ACM cases (Lombardi and Marian, 2010; Basso et al., 2011). Thus, ACM is considered a disease of desmosomes, structures responsible not only for myocytes-myocyte attachment but also hubs of molecular pathways regulated at the cell junctions. Indeed, it has been shown that desmosomal proteins are not only structural proteins but have also signaling functions as they regulate cellular proliferation, differentiation, apoptosis, and gene expression (Garcia-Gras et al., 2006; Bass-Zubek et al., 2009; Lombardi and Marian, 2010; Delmar and McKenna, 2010; Lombardi et al., 2011; Chen et al., 2014; Gurha et al., 2016; van Opbergen et al., 2019).

Animal and cellular models indicate abnormal biomechanical properties, and crosstalks from the desmosome to the cytoskeleton, nucleus, gap junctions, and ion channels are implicated in the pathobiology of ACM (Garcia-Gras et al., 2006; Bass-Zubek et al., 2009; Lombardi and Marian, 2010; Delmar and McKenna, 2010; Lombardi et al., 2011; Chen et al., 2014; Gurha et al., 2016; van Opbergen et al., 2019; Puzzi et al., 2019).

Fibrofatty infiltration is a common feature of SMD and ACM (Pearce et al., 1981; Hadar et al., 1983; Lombardi and Marian, 2010; Basso et al., 2011) and may be considered an anomaly of cell differentiation. This observation has prompted researchers to investigate the role of the canonical Wnt signaling and the Hippo pathway, two molecular pathways known to regulate embryonic development and adult tissue homeostasis, on the development of fibroadiposis in both conditions.

### Wnt Signaling in ACM and SMD

The Wnt signaling network controls embryonic development and adult tissue homeostasis, through the regulation of proliferation, cell polarity and migration, and cell fate specification (Komiya and Habas, 2008). It includes three highly conserved signaling pathways: the canonical β-catenin-dependent Wnt pathway and the two non-canonical β-catenin independent pathways (the non-canonical Wnt planar cell polarity and the non-canonical Wnt/calcium pathways) (Komiya and Habas, 2008). Wnt ligands are a family of secreted glycoproteins with autocrine and paracrine functions (Komiya and Habas, 2008).

Canonical and non-canonical Wnt pathways are known to compete between each other; the predominance of a pathway over the other depends on the expression of specific Wnt ligands and cell-surface receptors [Frizzled receptors (Fzd)] and coreceptors in a given cell or tissue, at a given time point (Grumolato et al., 2010; MacDonald and He, 2012).

β-Catenin is the main effector of the canonical Wnt signaling (Grumolato et al., 2010). In the absence of Wnt signals, β-catenin is incorporated in the so-called destruction complex where β-catenin is phosphorylated by the glycogen synthase kinase 3-beta (GSK3β) and degraded (Grumolato et al., 2010). Upon Wnt binding to Fzd receptors and the coreceptor Lrp5/6, the components of the destruction complex are recruited to the plasma membrane preventing the degradation of β-catenin that translocates to the nucleus and binds the TCF/LEF transcription factor (MacDonald et al., 2009; MacDonald and He, 2012).

Suppression of the canonical Wnt/β-catenin signaling is known to provoke adipogenesis, fibrogenesis, and apoptosis (Ross et al., 2000; Chen et al., 2001; Longo et al., 2002).

We have identified suppression of the canonical Wnt signaling as an important mechanism for the enhanced adipogenesis in desmosomal ACM (Garcia-Gras et al., 2006; Lombardi and Marian, 2010; Lombardi et al., 2011; Chen et al., 2014). We showed that mutations in desmosome genes alter assembly of the desmosomes and cause partial relocalization of JUP to the nucleus where it competes with β-catenin for binding to the transcription factor TCF/LEF, resulting in suppression of the canonical Wnt signaling (Garcia-Gras et al., 2006; Lombardi et al., 2011), which in turn determines a switch from myogenesis to adipogenesis in cardiac progenitors (Lombardi et al., 2009, 2011).

Inhibition of canonical Wnt signaling because of the activation of GSK3β and increased degradation of β-catenin has been also shown in a non-desmosomal form of ACM caused by a *TMEM43* mutation (Padron-Barthe et al., 2019). Another study in an ACM transgenic mouse model with cardiomyocyte-specific overexpression of a FLAG-tagged human desmoglein-2 harboring the Q558^∗^ nonsense mutation has confirmed inhibition of Wnt signaling in the pathogenesis of the disease (Calore et al., 2019). Moreover, a zebrafish model of DSP deficiency has been recently generated for *in vivo* cell signaling screen, using pathway-specific reporter transgenes. Out of nine considered, three pathways (Wnt/β-catenin, TGFβ/Smad3, and Hippo/YAP-TAZ) were significantly altered, with Wnt as the most dramatically affected (Giuliodori et al., 2018). The findings of all these papers point to Wnt/β-catenin as the final common pathway underlying the ACM pathogenesis, independently from the causal gene. The role of the nuclear translocation of plakoglobin as the main mechanism for the inhibition of Wnt signaling in ACM is still uncertain. A paper on a mouse model with cardiac-specific deletion of JUP showed that despite the model largely recapitulated the phenotype of human ACM, the Wnt/β-catenin-mediated signaling was not altered, while transforming growth factor-beta-mediated signaling was found significantly elevated (Li et al., 2011). Furthermore, a 2009 paper reported that endomyocardial biopsies from patients with ACM, but not controls, present with a marked reduction in immune-reactive signal levels for plakoglobin, suggesting that routine immunohistochemical analysis for plakoglobin expression of conventional endomyocardial biopsy samples could be a highly sensitive and specific diagnostic test for ACM (Asimaki et al., 2009). However, later on, subsequent studies revealed that plakoglobin-signal reduction was not a sensitive (Ermakov et al., 2014) nor a specific diagnostic marker for ACM, as it could also be demonstrated in other cardiac diseases, such as in sarcoidosis and giant-cell myocarditis (Asimaki et al., 2011).

Published data on the impact of Wnt signaling in the pathogenesis of muscular dystrophies have been conflicting. Increased β-catenin/Tcf transcriptional activity has been detected in the circulation, along with increased protein levels of β-catenin and enhanced DNA-binding activity of β-catenin/TCF in the skeletal muscle of DMD patients, suggestive of negative effects of activation of the canonical Wnt signaling pathway in DMD (Liu et al., 2016). On the contrary, activation of the canonical Wnt signaling pathway by intramuscular injection of Wnt3a in mdx mice has been proven to be beneficial as it attenuated the dystrophic phenotype (Shang et al., 2016). The inconsistence of these reports is most likely due to differences in the timing in which the Wnt signaling was assessed with regard to the state of cellular differentiation or to the interaction with other signaling pathways.

### Hippo Signaling in ACM and SMD

The Hippo signaling is an evolutionally conserved pathway consisting of a cascade of serine/threonine kinases: the tumor suppressor Hippo (MST1/2), large tumor suppressor kinases 1/2 (LATS1/2), and Yes-associated protein (YAP) (Pan, 2010; Halder and Johnson, 2011). YAP, the effector of Hippo pathway, is negatively regulated by phosphorylation by its upstream kinases. Non-phosphorylated/active YAP migrates to the nucleus, where it interacts with TEA domain family member (TEAD) transcription factor and regulates cell fate, muscle growth, regeneration, and wasting (Pan, 2010; Halder and Johnson, 2011; Zhou et al., 2015). So far, only limited studies have explored the relationship between the Hippo pathway and the pathogenesis of ACM or muscular dystrophies.

A study from our group in human hearts with ACM and two independent mouse and cell culture ACM models identified activation of the Hippo pathway as a major mechanism in the pathogenesis of ACM (Chen et al., 2014). We showed that, in ACM, mutations in genes encoding desmosome proteins, by impairing cell–cell attachment, activate neurofibromin 2 (NF2), the upstream molecule of the Hippo pathway. Active NF2 initiates the cascade of the Hippo kinases downstream to NF2, which culminates in YAP phosphorylation/inactivation. As a result, gene expression through TEAD is suppressed (Chen et al., 2014). In addition, we showed that activation of the Hippo pathway contributes to suppress the canonical Wnt signaling as phosphorylated YAP sequestrates β-catenin in the cytosol, preventing its translocation into the nucleus (Chen et al., 2014). Collectively, these findings provide a mechanistic link between the mutant desmosome protein and enhanced adipogenesis through the Hippo and the canonical Wnt signaling pathways in ACM. Activation of Hippo/YAP-TAZ in the pathogenesis of ACM has also been confirmed by a recent study on a novel zebrafish model of DSP deficiency (Giuliodori et al., 2018).

In the skeletal muscle, YAP has a major role in myoblast proliferation, atrophy/hypertrophy, and mechanotransduction (Judson et al., 2012; Wei et al., 2013; Fischer et al., 2016).

A reduction in active YAP protein expression and increased LATS1/2 kinase activity has been found in skeletal muscle specimens from DMD patients but not in muscles from patients with other types of muscular dystrophy (Vita et al., 2018). However, these results from DMD patients have not been completely reproduced in mdx mice in which, although the increase in LATS1/2 activity was confirmed, the levels of total YAP and phosphorylated YAP were found to be elevated or not changed (Vita et al., 2018). The findings suggest that activation of Hippo is implicated in the pathogenesis of DMD, but the specific functions of key molecular regulators remain largely unknown.

A recent paper has shown that, in cardiac myocytes, dystroglycan 1 (DAG1), a component of the DGC, directly binds to YAP and inhibits cardiomyocyte proliferation; moreover, Hippo-induced YAP phosphorylation promotes YAP–DAG1 interaction, suggesting cooperation between Hippo pathway and DGC in preventing the nuclear localization of YAP (Morikawa et al., 2017). In the same paper, the authors show that, in the absence of dystrophin (like in DMD), the interaction of YAP with DGC is disrupted (Morikawa et al., 2017); however, the mechanism through which phosphorylated YAP is sequestered to the cell membrane in the absence of dystrophin and sarcoglycan-δ is not identified.

### Membrane Channels and Calcium Signaling in ACM and SMD

Several studies suggest dysregulation of ion channels and of the Ca^2+^ signaling machinery in ACM, not only as direct effect of causal mutations located in genes encoding components of the Ca^2+^ cycling machinery (such as PLN) but also as a consequence of mutations in desmosomal genes (Noorman et al., 2013).

*In vitro* and *in vivo* studies have shown that decreased expression of PKP2 and DSP in cardiomyocytes affects expression levels, phosphorylation, and function of connexin 40 and connexin 43 independently of the cell–cell detachment and prior to the fibrofatty infiltration of the myocardium (Oxford et al., 2007; Noorman et al., 2013; Lyon et al., 2014). Additionally, mutations in desmosomal proteins have been shown to affect the sodium channel function prior to cardiomyopathic changes (Sato et al., 2009; Rizzo et al., 2012; Cerrone et al., 2014). These data suggest a role for desmosomal proteins as stabilizers of the gap junction integrity and highlight the molecular mechanisms of early electrical defects found in ACM patients.

ACM causal mutations in the non-desmosomal gene encoding PLN directly impair the calcium handling machinery (van der Zwaag et al., 2012; van der Zwaag et al., 2013). On the other hand, recent studies in induced pluripotent stem cell-derived cardiomyocytes (hIPSC-CMs) and mouse models have shown that cardiomyocyte PKP2 deficiency causes calcium handling dysregulation by affecting the transcription of genes and the function of components of intracellular calcium cycling machinery (Kim C. et al., 2013; Cerrone et al., 2017; Kim et al., 2019).

Abnormal calcium homeostasis has also been described in the pathogenesis of cardiac disease in the course of dystrophinopathies (Whitehead et al., 2006; Williams and Allen, 2007; Fanchaouy et al., 2009). The absence of dystrophin in the heart renders cardiomyocytes more sensitive to stretch-induced damage leading to loss of plasma membrane integrity, which results in an increased calcium influx into the cell (Yasuda et al., 2005; Yeung et al., 2005; Whitehead et al., 2006; Williams and Allen, 2007; Fanchaouy et al., 2009). Elevation in intracellular Ca^2+^ has several negative effects: mitochondrial deregulation, induction of protease calpain-mediated necrosis, activation of Ca^2+^/calmodulin (CaM) and CaM kinase II (CaMKII) and protein kinase A (PKA), and activation of nuclear factor kappa B (NF-κB) and of neuronal nitric oxide synthase (nNOS) (Yeung et al., 2005; Whitehead et al., 2006; Williams and Allen, 2007; Fanchaouy et al., 2009). The resulting cell damage causes degeneration of the cardiomyocytes and fibrosis, which are responsible of development of dilated cardiomyopathy.

### Cell Origin of Fibroadiposis in ACM and SMD

Skeletal muscle consists of fascicles of elongated multinucleate cells, called myofibers, while the myocardium is composed of single binucleate cardiomyocytes connected through the intercalated disks, unique structures that enable them to work as a single functional syncytium. Both adult skeletal and cardiac muscle cells do not divide; however, while skeletal muscle is able to have a considerable amount of regeneration, cardiac myocytes have a limited regeneration capacity after injury.

Upon skeletal muscle injury, satellite cells (SCs) promptly re-enter the cell-cycle, proliferate and differentiate in order to repair the damaged myofibers, and, at the same time, repopulate the SC reserve pool by self-renewing (Feige et al., 2018). On the other hand, myocyte death due to chronic cardiac injury is not followed by replacement with new cardiomyocytes but results in activation of the extracellular matrix and consequent fibrosis.

Genetic mutations in ACM and in SMD cause persistent muscle damage; the resulting progressive-chronic injury induces abnormal tissue repair ending in fibroadiposis.

In the skeletal muscle, effective repair requires functional crosstalk of SCs with other resident cell types including motor neurons, endothelial cells, immune cells, fibrogenic cells, and adipogenic precursors (Tatsumi et al., 2009; Uezumi et al., 2010, 2011; Heredia et al., 2013; Verma et al., 2018). Among them, mesenchymal progenitors identified by the surface marker PDGFRA have recently emerged as important players in skeletal muscle regeneration, but they have also been identified as the main source of intramuscular fibro/adipogenesis in pathological conditions (Joe et al., 2010; Uezumi et al., 2010, 2011). PDGFRα + cells are also known as fibroadipocyte progenitors (FAPs) because of their fibrogenic and adipogenic potential (Uezumi et al., 2010, 2011). Upon acute muscle injury, FAPs are activated and, in normal conditions, act in synergy with SCs to promote efficient muscle regeneration (Joe et al., 2010; Uezumi et al., 2010, 2011; Heredia et al., 2013). In contrast, when the muscle injury is persistent, like in muscular dystrophies, activated FAPs differentiate to fibroblasts and adipocytes (Joe et al., 2010; Uezumi et al., 2010, 2011; Heredia et al., 2013).

In ACM, the cell source of adipocytes has been an enigma for many years. Recently, our group has shown that cardiac progenitor cells (CPCs) from the second heart field are a source of adipocytes in ACM (Lombardi et al., 2009, 2011; Lombardi and Marian, 2010, 2011); however, these cells are rare in the heart and account for only a small fraction of the adipocytes. Thus, cardiac cells other than CPCs might contribute to fibroadiposis in ACM.

Since ACM and SMD show similar fibroadipocytic replacement of muscle, we surmised that the heart, like skeletal muscle, might contain resident FAPs, which could differentiate to adipocytes in the presence of chronic injury due to the presence of mutant desmosomal proteins. Therefore, we isolated from human and mouse heart a population of progenitor cells, positive for PDGFRA and negative for other lineage and fibroblast markers, which we named cardiac FAPs (Lombardi et al., 2016). Cardiac FAPs express desmosomal proteins (which are encoded by most of ACM causal genes) and are bipotential as the majority express the fibroblast marker collagen 1 α-1, while a small subset expresses the adipogenic marker CCAAT/enhancer-binding protein α (Lombardi et al., 2016). *In vivo* genetic fate-mapping experiments demonstrated ∼40% of adipocytes in the heart of a mouse model of ACM originates from FAPs, through a Wnt-dependent mechanism (Lombardi et al., 2016). The findings expand the cellular spectrum of ACM, commonly recognized as a disease of cardiomyocytes, to include non-myocyte cells in the heart.

## Conclusion

Arrhythmogenic cardiomyopathy and SMDs share many histological and molecular/cellular pathogenic mechanisms; for this reason, research findings from either pathology are expected to have significant reciprocal impact. Muscle degeneration, inflammation, and fibroadipocytic replacement have been described, and common molecular pathways (such as Wnt and Hippo signaling) and cell types have been shown to play a pathogenic role in both diseases. Moreover autoimmunity is an emerging research area with important translational promises for the clinical management and treatment of affected individual in both conditions.

In this review, we summarize for the first time the analogies between studies on skeletal muscle dystrophies and arrhythmogenic cardiomyopathy, with a particular focus on the findings with the highest potential for knowledge exchange between the two research fields.

## Author Contributions

SG, SC, and RL contributed to the preparation of the whole manuscript. CD wrote the MRI paragraph. All authors contributed to the article and approved the submitted version.

## Conflict of Interest

The authors declare that the research was conducted in the absence of any commercial or financial relationships that could be construed as a potential conflict of interest.

## References

[B1] Al-Sabeq B. Krahn A. D. Conacher S. Klein G. J. Laksman Z. (2014). Arrhythmogenic right ventricular cardiomyopathy with recessive inheritance related to a new homozygous desmocollin-2 mutation. *Can. J. Cardiol.* 30 e1–e3. 24793512 10.1016/j.cjca.2014.01.014

[B2] Arahata K. Engel A. G. (1984). Monoclonal antibody analysis of mononuclear cells in myopathies. I: quantitation of subsets according to diagnosis and sites of accumulation and demonstration and counts of muscle fibers invaded by T cells. *Ann. Neurol.* 16 193–208. 10.1002/ana.410160206 6383191

[B3] Asimaki A. Tandri H. Duffy E. R. Winterfield J. R. Mackey-Bojack S. Picken M. M. (2011). Altered desmosomal proteins in granulomatous myocarditis and potential pathogenic links to arrhythmogenic right ventricular cardiomyopathy. *Circ. Arrhythm. Electrophysiol.* 4 743–752. 10.1161/circep.111.964890 21859801 PMC3203520

[B4] Asimaki A. Tandri H. Huang H. Halushka M. K. Gautam S. Basso C. (2009). A new diagnostic test for arrhythmogenic right ventricular cardiomyopathy. *N Engl. J. Med.* 360 1075–1084. 19279339 10.1056/NEJMoa0808138

[B5] Barros Maranhao J. de Oliveira Moreira D. Mauricio A. F. de Carvalho S. C. Ferretti R. Pereira J. A. (2015). Changes in calsequestrin, TNF-alpha, TGF-beta and MyoD levels during the progression of skeletal muscle dystrophy in mdx mice: a comparative analysis of the quadriceps, diaphragm and intrinsic laryngeal muscles. *Int. J. Exp. Pathol.* 96 285–293. 10.1111/iep.12142 26515458 PMC4693553

[B6] Basso C. Bauce B. Corrado D. Thiene G. (2011). Pathophysiology of arrhythmogenic cardiomyopathy. *Nat. Rev. Cardiol.* 9 223–233. 10.1038/nrcardio.2011.173 22124316

[B7] Basso C. Corrado D. Thiene G. (2010). Arrhythmogenic right ventricular cardiomyopathy: what’s in a name? From a congenital defect (dysplasia) to a genetically determined cardiomyopathy (dystrophy). *Am. J. Cardiol.* 106 275–277. 10.1016/j.amjcard.2010.03.055 20599015

[B8] Basso C. Ronco F. Marcus F. Abudureheman A. Rizzo S. Frigo A. C. (2008). Quantitative assessment of endomyocardial biopsy in arrhythmogenic right ventricular cardiomyopathy/dysplasia: an in vitro validation of diagnostic criteria. *Eur. Heart J.* 29 2760–2771. 10.1093/eurheartj/ehn415 18819962

[B9] Basso C. Thiene G. Corrado D. Angelini A. Nava A. Valente M. (1996). Arrhythmogenic right ventricular cardiomyopathy. Dysplasia, dystrophy, or myocarditis? *Circulation* 94 983–991. 10.1161/01.cir.94.5.983 8790036

[B10] Bass-Zubek A. E. Godsel L. M. Delmar M. Green K. J. (2009). Plakophilins: multifunctional scaffolds for adhesion and signaling. *Curr. Opin. Cell. Biol.* 21 708–716. 10.1016/j.ceb.2009.07.002 19674883 PMC3091506

[B11] Beffagna G. Occhi G. Nava A. Vitiello L. Ditadi A. Basso C. (2005). Regulatory mutations in transforming growth factor-beta3 gene cause arrhythmogenic right ventricular cardiomyopathy type 1. *Cardiovasc. Res.* 65 366–373. 10.1016/j.cardiores.2004.10.005 15639475

[B12] Bermudez-Jimenez F. J. Carriel V. Brodehl A. Alaminos M. Campos A. Schirmer I. (2018). novel desmin mutation p.Glu401Asp impairs filament formation, disrupts cell membrane integrity, and causes severe arrhythmogenic left ventricular cardiomyopathy/dysplasia. *Circulation* 137 1595–1610. 10.1161/circulationaha.117.028719 29212896

[B13] Brun F. Gigli M. Graw S. L. Judge D. P. Merlo M. Murray B. (2020). FLNC truncations cause arrhythmogenic right ventricular cardiomyopathy. *J. Med. Genet.* 57 254–257. 10.1136/jmedgenet-2019-106394 31924696 PMC7539291

[B14] Burzyn D. Kuswanto W. Kolodin D. Shadrach J. L. Cerletti M. Jang Y. (2013). A special population of regulatory T cells potentiates muscle repair. *Cell* 155 1282–1295. 10.1016/j.cell.2013.10.054 24315098 PMC3894749

[B15] Caforio A. L. Keeling P. J. Zachara E. Mestroni L. Camerini F. Mann J. M. (1994). Evidence from family studies for autoimmunity in dilated cardiomyopathy. *Lancet* 344 773–777. 10.1016/s0140-6736(94)92339-6 7916071

[B16] Caforio A. L. P. Re F. Avella A. Marcolongo R. Baratta P. Seguso M. (2020). Evidence from family studies for autoimmunity in arrhythmogenic right ventricular cardiomyopathy: associations of circulating anti-heart and anti-intercalated disk autoantibodies with disease severity and family history. *Circulation* 141 1238–1248. 10.1161/circulationaha.119.043931 32114801

[B17] Calore M. Lorenzon A. Vitiello L. Poloni G. Khan M. A. F. Beffagna G. (2019). A novel murine model for arrhythmogenic cardiomyopathy points to a pathogenic role of Wnt signalling and miRNA dysregulation. *Cardiovasc. Res.* 115 739–751. 10.1093/cvr/cvy253 30304392

[B18] Campian M. E. Verberne H. J. Hardziyenka M. de Groot E. A. van Moerkerken A. F. van Eck-Smit B. L. (2010). Assessment of inflammation in patients with arrhythmogenic right ventricular cardiomyopathy/dysplasia. *Eur. J. Nucl. Med. Mol. Imaging.* 37 2079–2085. 20603720 10.1007/s00259-010-1525-y PMC2948173

[B19] Capell B. C. Collins F. S. (2006). Human laminopathies: nuclei gone genetically awry. *Nat. Rev. Genet.* 7 940–952. 10.1038/nrg1906 17139325

[B20] Cerrone M. Lin X. Zhang M. Agullo-Pascual E. Pfenniger A. Chkourko Gusky H. (2014). Missense mutations in plakophilin-2 cause sodium current deficit and associate with a Brugada syndrome phenotype. *Circulation* 129 1092–1103. 10.1161/circulationaha.113.003077 24352520 PMC3954430

[B21] Cerrone M. Montnach J. Lin X. Zhao Y. T. Zhang M. Agullo-Pascual E. (2017). Plakophilin-2 is required for transcription of genes that control calcium cycling and cardiac rhythm. *Nat. Commun.* 8:106. 10.1038/s41467-017-00127-0 PMC5524637 28740174

[B22] Chatterjee D. Fatah M. Akdis D. Spears D. A. Koopmann T. T. Mittal K. (2018). An autoantibody identifies arrhythmogenic right ventricular cardiomyopathy and participates in its pathogenesis. *Eur. Heart J.* 39 3932–3944. 10.1093/eurheartj/ehy567 30239670 PMC6247665

[B23] Cheedipudi S. M. Matkovich S. J. Coarfa C. Hu X. Robertson M. J. Sweet M. (2019). Genomic reorganization of lamin-associated domains in cardiac myocytes is associated with differential gene expression and DNA methylation in human dilated cardiomyopathy. *Circ. Res.* 124 1198–1213. 10.1161/circresaha.118.314177 30739589 PMC6459729

[B24] Chen S. Guttridge D. C. You Z. Zhang Z. Fribley A. Mayo M. W. (2001). Wnt-1 signaling inhibits apoptosis by activating beta-catenin/T cell factor-mediated transcription. *J. Cell Biol.* 152 87–96. 10.1083/jcb.152.1.87 11149923 PMC2193656

[B25] Chen S. N. Gurha P. Lombardi R. Ruggiero A. Willerson J. T. Marian A. J. (2014). The hippo pathway is activated and is a causal mechanism for adipogenesis in arrhythmogenic cardiomyopathy. *Circ. Res.* 114 454–468. 10.1161/circresaha.114.302810 24276085 PMC3946717

[B26] Chen S. N. Lombardi R. Karmouch J. Tsai J. Y. Czernuszewicz G. Taylor M. R. G. (2019). DNA damage response/TP53 pathway is activated and contributes to the pathogenesis of dilated cardiomyopathy associated with LMNA (Lamin A/C) mutations. *Circ. Res.* 124 856–873. 10.1161/circresaha.118.314238 30696354 PMC6460911

[B27] Consalvi S. Mozzetta C. Bettica P. Germani M. Fiorentini F. Del Bene F. (2013). Preclinical studies in the mdx mouse model of duchenne muscular dystrophy with the histone deacetylase inhibitor givinostat. *Mol. Med.* 19 79–87. 10.2119/molmed.2013.00011 23552722 PMC3667212

[B28] Corrado D. Basso C. Thiene G. McKenna W. J. Davies M. J. Fontaliran F. (1997). Spectrum of clinicopathologic manifestations of arrhythmogenic right ventricular cardiomyopathy/dysplasia: a multicenter study. *J. Am. Coll. Cardiol.* 30 1512–1520. 10.1016/s0735-1097(97)00332-x 9362410

[B29] Cruz-Guzman Odel R. Rodriguez-Cruz M. Escobar Cedillo R. E. (2015). Systemic inflammation in duchenne muscular dystrophy: association with muscle function and nutritional status. *Biomed Res Int.* 2015:891972. 10.1155/2015/891972 PMC4561314 26380303

[B30] De Deyne S. De la Gastine B. Gras G. Dargere S. Verdon R. Coquerel A. (2006). Acute renal failure with acyclovir in a 42-year-old patient without previous renal dysfunction. *Rev. Med. Interne* 27 892–894. 16854507 10.1016/j.revmed.2006.06.008

[B31] Delmar M. McKenna W. J. (2010). The cardiac desmosome and arrhythmogenic cardiomyopathies: from gene to disease. *Circ. Res.* 107 700–714. 10.1161/circresaha.110.223412 20847325

[B32] den Haan A. D. Tan B. Y. Zikusoka M. N. Llado L. I. Jain R. Daly A. (2009). Comprehensive desmosome mutation analysis in north americans with arrhythmogenic right ventricular dysplasia/cardiomyopathy. *Circ. Cardiovasc. Genet.* 2 428–435. 10.1161/circgenetics.109.858217 20031617 PMC2801867

[B33] Elliott P. M. Anastasakis A. Asimaki A. Basso C. Bauce B. Brooke M. A. (2019). Definition and treatment of arrhythmogenic cardiomyopathy: an updated expert panel report. *Eur. J. Heart Fail.* 21 955–964. 10.1002/ejhf.1534 31210398 PMC6685753

[B34] Ermakov S. Ursell P. C. Johnson C. J. Meadows A. Zhao S. Marcus G. M. (2014). Plakoglobin immunolocalization as a diagnostic test for arrhythmogenic right ventricular cardiomyopathy. *PACE* 37 1708–1716. 10.1111/pace.12492 25196244

[B35] Ervasti J. M. Campbell K. P. (1993). A role for the dystrophin-glycoprotein complex as a transmembrane linker between laminin and actin. *J. Cell Biol.* 122 809–823. 8349731 10.1083/jcb.122.4.809 PMC2119587

[B36] Fanchaouy M. Polakova E. Jung C. Ogrodnik J. Shirokova N. Niggli E. (2009). Pathways of abnormal stress-induced Ca2+ influx into dystrophic mdx cardiomyocytes. *Cell Calcium.* 46 114–121. 19604578 10.1016/j.ceca.2009.06.002 PMC2745084

[B37] Feige P. Brun C. E. Ritso M. Rudnicki M. A. (2018). Orienting muscle stem cells for regeneration in homeostasis. aging, and disease. *Cell Stem. Cell.* 23 653–664. 30388423 10.1016/j.stem.2018.10.006 PMC6262894

[B38] Finanger E. L. Russman B. Forbes S. C. Rooney W. D. Walter G. A. Vandenborne K. (2012). Use of skeletal muscle MRI in diagnosis and monitoring disease progression in Duchenne muscular dystrophy. *Phys. Med. Rehabil. Clin. N. Am.* 23 1–10. 22239869 10.1016/j.pmr.2011.11.004 PMC3561672

[B39] Fischer M. Rikeit P. Knaus P. Coirault C. Y. A. P. - (2016). Mediated mechanotransduction in skeletal muscle. *Front. Physiol.* 7:41. 10.3389/fphys.2016.00041 26909043 PMC4754448

[B40] Flanigan K. M. Campbell K. Viollet L. Wang W. Gomez A. M. Walker C. M. (2013). Anti-dystrophin T cell responses in Duchenne muscular dystrophy: prevalence and a glucocorticoid treatment effect. *Hum. Gene Ther.* 24 797–806. 24010700 10.1089/hum.2013.092 PMC3768239

[B41] Garcia-Gras E. Lombardi R. Giocondo M. J. Willerson J. T. Schneider M. D. Khoury D. S. (2006). Suppression of canonical Wnt/beta-catenin signaling by nuclear plakoglobin recapitulates phenotype of arrhythmogenic right ventricular cardiomyopathy. *J. Clin. Invest.* 116 2012–2021. 16823493 10.1172/JCI27751 PMC1483165

[B42] Garrood P. Hollingsworth K. G. Eagle M. Aribisala B. S. Birchall D. Bushby K. (2009). MR imaging in Duchenne muscular dystrophy: quantification of T1-weighted signal, contrast uptake, and the effects of exercise. *J. Magn. Reson. Imaging.* 30 1130–1138. 19856446 10.1002/jmri.21941

[B43] Gehmlich K. Asimaki A. Cahill T. J. Ehler E. Syrris P. Zachara E. (2010). Novel missense mutations in exon 15 of desmoglein-2: role of the intracellular cadherin segment in arrhythmogenic right ventricular cardiomyopathy? *Heart Rhythm.* 7 1446–1453. 20708101 10.1016/j.hrthm.2010.08.007 PMC2994644

[B44] Gerull B. Heuser A. Wichter T. Paul M. Basson C. T. McDermott D. A. (2004). Mutations in the desmosomal protein plakophilin-2 are common in arrhythmogenic right ventricular cardiomyopathy. *Nat Genet.* 36 1162–1164. 15489853 10.1038/ng1461

[B45] Giuliodori A. Beffagna G. Marchetto G. Fornetto C. Vanzi F. Toppo S. (2018). Loss of cardiac Wnt/beta-catenin signalling in desmoplakin-deficient AC8 zebrafish models is rescuable by genetic and pharmacological intervention. *Cardiovasc. Res.* 114 1082–1097. 29522173 10.1093/cvr/cvy057

[B46] Grumolato L. Liu G. Mong P. Mudbhary R. Biswas R. Arroyave R. (2010). Canonical and noncanonical Wnts use a common mechanism to activate completely unrelated coreceptors. *Genes Dev.* 24 2517–2530. 21078818 10.1101/gad.1957710 PMC2975928

[B47] Gurha P. Chen X. Lombardi R. Willerson J. T. Marian A. J. (2016). Knockdown of plakophilin 2 downregulates miR-184 through CpG hypermethylation and suppression of the E2F1 pathway and leads to enhanced adipogenesis in vitro. *Circ. Res.* 119 731–750. 27470638 10.1161/CIRCRESAHA.116.308422 PMC5010490

[B48] Gussoni E. Pavlath G. K. Miller R. G. Panzara M. A. Powell M. Blau H. M. (1994). Specific T cell receptor gene rearrangements at the site of muscle degeneration in Duchenne muscular dystrophy. *J. Immunol.* 153 4798–4805. 7963545

[B49] Hackman P. Marchand S. Sarparanta J. Vihola A. Penisson-Besnier I. Eymard B. (2008). Truncating mutations in C-terminal titin may cause more severe tibial muscular dystrophy (TMD). *Neuromuscul. Disord.* 18 922–928. 18948003 10.1016/j.nmd.2008.07.010

[B50] Hackman P. Vihola A. Haravuori H. Marchand S. Sarparanta J. De Seze J. (2002). Tibial muscular dystrophy is a titinopathy caused by mutations in TTN, the gene encoding the giant skeletal-muscle protein titin. *Am. J. Hum. Genet.* 71 492–500. 12145747 10.1086/342380 PMC379188

[B51] Hadar H. Gadoth N. Heifetz M. (1983). Fatty replacement of lower paraspinal muscles: normal and neuromuscular disorders. *AJR Am. J. Roentgenol.* 141 895–898. 6605058 10.2214/ajr.141.5.895

[B52] Hagenbuch S. C. Gottliebson W. M. Wansapura J. Mazur W. Fleck R. Benson D. W. (2010). Detection of progressive cardiac dysfunction by serial evaluation of circumferential strain in patients with Duchenne muscular dystrophy. *Am. J. Cardiol.* 105 1451–1455. 20451693 10.1016/j.amjcard.2009.12.070

[B53] Halder G. Johnson R. L. (2011). Hippo signaling: growth control and beyond. *Development* 138 9–22. 21138973 10.1242/dev.045500 PMC2998162

[B54] Hall C. L. Akhtar M. M. Sabater-Molina M. Futema M. Asimaki A. Protonotarios A. (2019). Filamin C variants are associated with a distinctive clinical and immunohistochemical arrhythmogenic cardiomyopathy phenotype. *Int. J. Cardiol.* 307 101–108. 31627847 10.1016/j.ijcard.2019.09.048

[B55] Hedberg C. Melberg A. Kuhl A. Jenne D. Oldfors A. (2012). Autosomal dominant myofibrillar myopathy with arrhythmogenic right ventricular cardiomyopathy 7 is caused by a DES mutation. *Eur. J. Hum. Genet.* 20 984–985. 22395865 10.1038/ejhg.2012.39 PMC3421124

[B56] Heredia J. E. Mukundan L. Chen F. M. Mueller A. A. Deo R. C. Locksley R. M. (2013). Type 2 innate signals stimulate fibro/adipogenic progenitors to facilitate muscle regeneration. *Cell* 153 376–388. 23582327 10.1016/j.cell.2013.02.053 PMC3663598

[B57] Hiser W. (2020). *Duchenne Muscular Dystrophy*. Available online at: http://www.pathologyoutlines.com/topic/muscleduchennemusculardystrophy.html

[B58] Hussein M. R. Hamed S. A. Mostafa M. G. Abu-Dief E. E. Kamel N. F. Kandil M. R. (2006). The effects of glucocorticoid therapy on the inflammatory and dendritic cells in muscular dystrophies. *Int. J. Exp. Pathol.* 87 451–461. 17222213 10.1111/j.1365-2613.2006.00470.x PMC2517389

[B59] Jefferies J. L. Eidem B. W. Belmont J. W. Craigen W. J. Ware S. M. Fernbach S. D. (2005). Genetic predictors and remodeling of dilated cardiomyopathy in muscular dystrophy. *Circulation* 112 2799–2804. 16246949 10.1161/CIRCULATIONAHA.104.528281

[B60] Joe A. W. Yi L. Natarajan A. Le Grand F. So L. Wang J. (2010). Muscle injury activates resident fibro/adipogenic progenitors that facilitate myogenesis. *Nat. Cell. Biol.* 12 153–163. 20081841 10.1038/ncb2015 PMC4580288

[B61] Judson R. N. Tremblay A. M. Knopp P. White R. B. Urcia R. De Bari C. (2012). The Hippo pathway member Yap plays a key role in influencing fate decisions in muscle satellite cells. *J. Cell Sci.* 125(Pt 24), 6009–6019. 23038772 10.1242/jcs.109546 PMC3585517

[B62] Kamdar F. Garry D. J. (2016). Dystrophin-deficient cardiomyopathy. *J. Am. Coll. Cardiol.* 67 2533–2546. 27230049 10.1016/j.jacc.2016.02.081

[B63] Kim C. Wong J. Wen J. Wang S. Wang C. Spiering S. (2013). Studying arrhythmogenic right ventricular dysplasia with patient-specific iPSCs. *Nature* 494 105–110. 23354045 10.1038/nature11799 PMC3753229

[B64] Kim H. K. Merrow A. C. Shiraj S. Wong B. L. Horn P. S. Laor T. (2013). Analysis of fatty infiltration and inflammation of the pelvic and thigh muscles in boys with Duchenne muscular dystrophy (DMD): grading of disease involvement on MR imaging and correlation with clinical assessments. *Pediatr. Radiol.* 43 1327–1335. 23666207 10.1007/s00247-013-2696-z

[B65] Kim J. C. Perez-Hernandez M. Alvarado F. J. Maurya S. R. Montnach J. Yin Y. (2019). Disruption of Ca(2+)i homeostasis and connexin 43 hemichannel function in the right ventricle precedes overt arrhythmogenic cardiomyopathy in plakophilin-2-deficient mice. *Circulation* 140 1015–1030. 31315456 10.1161/CIRCULATIONAHA.119.039710 PMC6746608

[B66] Klauke B. Kossmann S. Gaertner A. Brand K. Stork I. Brodehl A. (2010). De novo desmin-mutation N116S is associated with arrhythmogenic right ventricular cardiomyopathy. *Hum. Mol. Genet.* 19 4595–4607. 20829228 10.1093/hmg/ddq387

[B67] Komiya Y. Habas R. (2008). Wnt signal transduction pathways. *Organogenesis* 4 68–75. 19279717 10.4161/org.4.2.5851 PMC2634250

[B68] Kramer C. M. (2015). Role of Cardiac MR Imaging in cardiomyopathies. journal of nuclear medicine : official publication. *Soc. Nuclear Med.* 56 (Suppl. 4), 39S–45S. 10.2967/jnumed.114.142729 PMC4465292 26033902

[B69] Li J. Swope D. Raess N. Cheng L. Muller E. J. Radice G. L. (2011). Cardiac tissue-restricted deletion of plakoglobin results in progressive cardiomyopathy and activation of {beta}-catenin signaling. *Mol. Cell Biol.* 31 1134–1144. 21245375 10.1128/MCB.01025-10 PMC3067899

[B70] Liang W. C. Mitsuhashi H. Keduka E. Nonaka I. Noguchi S. Nishino I. (2011). TMEM43 mutations in Emery-Dreifuss muscular dystrophy-related myopathy. *Ann. Neurol.* 69 1005–1013. 21391237 10.1002/ana.22338

[B71] Liu F. Liang Z. Xu J. Li W. Zhao D. Zhao Y. (2016). Activation of the wnt/beta-catenin signaling pathway in polymyositis, dermatomyositis and Duchenne muscular dystrophy. *J. Clin. Neurol.* 12 351–360. 27165423 10.3988/jcn.2016.12.3.351 PMC4960221

[B72] Lombardi R. Chen S. N. Ruggiero A. Gurha P. Czernuszewicz G. Z. Willerson J. T. (2016). Cardiac fibro-adipocyte progenitors express desmosome proteins and preferentially differentiate to adipocytes upon deletion of the desmoplakin gene. *Circ. Res.* 119 41–54. 27121621 10.1161/CIRCRESAHA.115.308136 PMC4920717

[B73] Lombardi R. da Graca Cabreira-Hansen M. Bell A. Fromm R. R. Willerson J. T. Marian A. J. (2011). Nuclear plakoglobin is essential for differentiation of cardiac progenitor cells to adipocytes in arrhythmogenic right ventricular cardiomyopathy. *Circ. Res.* 109 1342–1353. 22021931 10.1161/CIRCRESAHA.111.255075 PMC3237769

[B74] Lombardi R. Dong J. Rodriguez G. Bell A. Leung T. K. Schwartz R. J. (2009). Genetic fate mapping identifies second heart field progenitor cells as a source of adipocytes in arrhythmogenic right ventricular cardiomyopathy. *Circ. Res.* 104 1076–1084. 19359597 10.1161/CIRCRESAHA.109.196899 PMC2767296

[B75] Lombardi R. Marian A. J. (2010). Arrhythmogenic right ventricular cardiomyopathy is a disease of cardiac stem cells. *Curr. Opin. Cardiol.* 25 222–228. 20124997 10.1097/HCO.0b013e3283376daf PMC2980568

[B76] Lombardi R. Marian A. J. (2011). Molecular genetics and pathogenesis of arrhythmogenic right ventricular cardiomyopathy: a disease of cardiac stem cells. *Pediatr. Cardiol.* 32 360–365. 21267716 10.1007/s00246-011-9890-2

[B77] Longo K. A. Kennell J. A. Ochocinska M. J. Ross S. E. Wright W. S. MacDougald O. A. (2002). Wnt signaling protects 3T3-L1 preadipocytes from apoptosis through induction of insulin-like growth factors. *J. Biol. Chem.* 277 38239–38244. 12154096 10.1074/jbc.M206402200

[B78] Lozanoska-Ochser B. Benedetti A. Rizzo G. Marrocco V. Di Maggio R. Fiore P. (2018). Targeting early PKCtheta-dependent T-cell infiltration of dystrophic muscle reduces disease severity in a mouse model of muscular dystrophy. *J. Pathol.* 244 323–333. 29214629 10.1002/path.5016

[B79] Lyon R. C. Mezzano V. Wright A. T. Pfeiffer E. Chuang J. Banares K. (2014). Connexin defects underlie arrhythmogenic right ventricular cardiomyopathy in a novel mouse model. *Hum. Mol. Genet.* 23 1134–1150. 24108106 10.1093/hmg/ddt508 PMC3919010

[B80] MacDonald B. T. He X. (2012). Frizzled and LRP5/6 receptors for Wnt/beta-catenin signaling. *Cold Spring Harb. Perspect.Biol.* 4:a007880. 10.1101/cshperspect.a007880 PMC3504444 23209147

[B81] MacDonald B. T. Tamai K. He X. (2009). Wnt/beta-catenin signaling: components, mechanisms, and diseases. *Dev. Cell* 17 9–26. 19619488 10.1016/j.devcel.2009.06.016 PMC2861485

[B82] Malecova B. Gatto S. Etxaniz U. Passafaro M. Cortez A. Nicoletti C. (2018). Dynamics of cellular states of fibro-adipogenic progenitors during myogenesis and muscular dystrophy. *Nat. Commun.* 9 3670. 30202063 10.1038/s41467-018-06068-6 PMC6131350

[B83] Mallat Z. Tedgui A. Fontaliran F. Frank R. Durigon M. Fontaine G. (1996). Evidence of apoptosis in arrhythmogenic right ventricular dysplasia. *N. Engl. J. Med.* 335 1190–1196. 8815941 10.1056/NEJM199610173351604

[B84] Marcus F. I. Fontaine G. H. Guiraudon G. Frank R. Laurenceau J. L. Malergue C. (1982). Right ventricular dysplasia: a report of 24 adult cases. *Circulation* 65 384–398. 7053899 10.1161/01.cir.65.2.384

[B85] Marcus F. I. McKenna W. J. Sherrill D. Basso C. Bauce B. Bluemke D. A. (2010). Diagnosis of arrhythmogenic right ventricular cardiomyopathy/dysplasia: proposed modification of the task force criteria. *Eur. Heart J.* 31 806–814. 20172912 10.1093/eurheartj/ehq025 PMC2848326

[B86] Matsumura K. Nakano I. Fukuda N. Ikehira H. Tateno Y. Aoki Y. (1988). Proton spin-lattice relaxation time of Duchenne dystrophy skeletal muscle by magnetic resonance imaging. *Muscle Nerve* 11 97–102. 3343998 10.1002/mus.880110202

[B87] Mayosi B. M. Fish M. Shaboodien G. Mastantuono E. Kraus S. Wieland T. (2017). Identification of cadherin 2 (CDH2) mutations in arrhythmogenic right ventricular cardiomyopathy. *Circ. Cardiovasc. Genet.* 10:e001605. 10.1161/CIRCGENETICS.116.001605 28280076

[B88] McKoy G. Protonotarios N. Crosby A. Tsatsopoulou A. Anastasakis A. Coonar A. (2000). Identification of a deletion in plakoglobin in arrhythmogenic right ventricular cardiomyopathy with palmoplantar keratoderma and woolly hair (Naxos disease). *Lancet.* 355 2119–2124. 10902626 10.1016/S0140-6736(00)02379-5

[B89] Melberg A. Oldfors A. Blomstrom-Lundqvist C. Stalberg E. Carlsson B. Larrson E. (1999). Autosomal dominant myofibrillar myopathy with arrhythmogenic right ventricular cardiomyopathy linked to chromosome 10q. *Ann. Neurol.* 46 684–692. 10970245 10.1002/1531-8249(199911)46:5<684::aid-ana2>3.0.co;2-#

[B90] Mendell J. R. Campbell K. Rodino-Klapac L. Sahenk Z. Shilling C. Lewis S. (2010). Dystrophin immunity in Duchenne’s muscular dystrophy. *N. Engl. J. Med.* 363 1429–1437. 20925545 10.1056/NEJMoa1000228 PMC3014106

[B91] Merner N. D. Hodgkinson K. A. Haywood A. F. Connors S. French V. M. Drenckhahn J. D. (2008). Arrhythmogenic right ventricular cardiomyopathy type 5 is a fully penetrant, lethal arrhythmic disorder caused by a missense mutation in the TMEM43 gene. *Am. J. Hum. Genet.* 82 809–821. 18313022 10.1016/j.ajhg.2008.01.010 PMC2427209

[B92] Miles C. Finocchiaro G. Papadakis M. Gray B. Westaby J. Ensam B. (2019). Sudden death and left ventricular involvement in arrhythmogenic cardiomyopathy. *Circulation* 139 1786–1797. 30700137 10.1161/CIRCULATIONAHA.118.037230 PMC6467560

[B93] Misaka T. Yoshihisa A. Takeishi Y. (2019). Titin in muscular dystrophy and cardiomyopathy: urinary titin as a novel marker. *Clin. Chim. Acta* 495 123–128. 30959043 10.1016/j.cca.2019.04.005

[B94] Monaco A. P. Bertelson C. J. Liechti-Gallati S. Moser H. Kunkel L. M. (1988). An explanation for the phenotypic differences between patients bearing partial deletions of the DMD locus. *Genomics* 2 90–95. 3384440 10.1016/0888-7543(88)90113-9

[B95] Morikawa Y. Heallen T. Leach J. Xiao Y. Martin J. F. (2017). Dystrophin-glycoprotein complex sequesters Yap to inhibit cardiomyocyte proliferation. *Nature* 547 227–231. 28581498 10.1038/nature22979 PMC5528853

[B96] Mukai T. Mori-Yoshimura M. Nishikawa A. Hokkoku K. Sonoo M. Nishino I. (2019). Emery-Dreifuss muscular dystrophy-related myopathy with TMEM43 mutations. *Muscle Nerve* 59 E5–E7. 30311943 10.1002/mus.26355

[B97] Nava A. Bauce B. Basso C. Muriago M. Rampazzo A. Villanova C. (2000). Clinical profile and long-term follow-up of 37 families with arrhythmogenic right ventricular cardiomyopathy. *J. Am. Coll. Cardiol.* 36 2226–2233. 11127465 10.1016/s0735-1097(00)00997-9

[B98] Nava A. Thiene G. Canciani B. Scognamiglio R. Daliento L. Buja G. (1988). Familial occurrence of right ventricular dysplasia: a study involving nine families. *J. Am. Coll. Cardiol.* 12 1222–1228. 3170963 10.1016/0735-1097(88)92603-4

[B99] Noorman M. Hakim S. Kessler E. Groeneweg J. A. Cox M. G. Asimaki A. (2013). Remodeling of the cardiac sodium channel, connexin43, and plakoglobin at the intercalated disk in patients with arrhythmogenic cardiomyopathy. *Heart Rhythm* 10 412–419. 23178689 10.1016/j.hrthm.2012.11.018 PMC3608196

[B100] Norgett E. E. Hatsell S. J. Carvajal-Huerta L. Cabezas J. C. Common J. Purkis P. E. (2000). Recessive mutation in desmoplakin disrupts desmoplakin-intermediate filament interactions and causes dilated cardiomyopathy, woolly hair and keratoderma. *Hum. Mol. Genet.* 9 2761–2766. 11063735 10.1093/hmg/9.18.2761

[B101] Nowak K. J. Davies K. E. (2004). Duchenne muscular dystrophy and dystrophin: pathogenesis and opportunities for treatment. *EMBO Rep.* 5 872–876. 15470384 10.1038/sj.embor.7400221 PMC1299132

[B102] Ortiz-Genga M. F. Cuenca S. Dal Ferro M. Zorio E. Salgado-Aranda R. Climent V. (2016). Truncating FLNC mutations are associated with high-risk dilated and arrhythmogenic cardiomyopathies. *J. Am. Coll. Cardiol.* 68 2440–2451. 27908349 10.1016/j.jacc.2016.09.927

[B103] Oxford E. M. Musa H. Maass K. Coombs W. Taffet S. M. Delmar M. (2007). Connexin43 remodeling caused by inhibition of plakophilin-2 expression in cardiac cells. *Circ. Res.* 101 703–711. 17673670 10.1161/CIRCRESAHA.107.154252

[B104] Padron-Barthe L. Villalba-Orero M. Gomez-Salinero J. M. Dominguez F. Roman M. Larrasa-Alonso J. (2019). Severe cardiac dysfunction and death caused by arrhythmogenic right ventricular cardiomyopathy type 5 are improved by inhibition of glycogen synthase kinase-3beta. *Circulation* 140 1188–1204. 31567019 10.1161/CIRCULATIONAHA.119.040366 PMC6784777

[B105] Pan D. (2010). The hippo signaling pathway in development and cancer. *Dev. Cell.* 19 491–505. 20951342 10.1016/j.devcel.2010.09.011 PMC3124840

[B106] Parsai C. O’Hanlon R. Prasad S. K. Mohiaddin R. H. (2012). Diagnostic and prognostic value of cardiovascular magnetic resonance in non-ischaemic cardiomyopathies. *J. Cardiovasc. Magn. Reson.* 14:54. 10.1186/1532-429X-14-54 PMC3436728 22857649

[B107] Patel A. R. Kramer C. M. (2017). Role of cardiac magnetic resonance in the diagnosis and prognosis of nonischemic cardiomyopathy. *JACC Cardiovasc. Imaging* 10(10 Pt A), 1180–1193. 28982571 10.1016/j.jcmg.2017.08.005 PMC5708889

[B108] Pearce P. H. Johnsen R. D. Wysocki S. J. Kakulas B. A. (1981). Muscle lipids in Duchenne muscular dystrophy. *Aust. J. Exp. Biol. Med. Sci.* 59 77–90. 7236123 10.1038/icb.1981.4

[B109] Pelosi L. Berardinelli M. G. Forcina L. Spelta E. Rizzuto E. Nicoletti C. (2015). Increased levels of interleukin-6 exacerbate the dystrophic phenotype in mdx mice. *Hum. Mol. Genet.* 24 6041–6053. 26251044 10.1093/hmg/ddv323 PMC4599671

[B110] Pilichou K. Nava A. Basso C. Beffagna G. Bauce B. Lorenzon A. (2006). Mutations in desmoglein-2 gene are associated with arrhythmogenic right ventricular cardiomyopathy. *Circulation* 113 1171–1179. 16505173 10.1161/CIRCULATIONAHA.105.583674

[B111] Pilichou K. Remme C. A. Basso C. Campian M. E. Rizzo S. Barnett P. (2009). Myocyte necrosis underlies progressive myocardial dystrophy in mouse dsg2-related arrhythmogenic right ventricular cardiomyopathy. *J. Exp. Med.* 206 1787–1802. 19635863 10.1084/jem.20090641 PMC2722163

[B112] Pollazzon M. Suominen T. Penttila S. Malandrini A. Carluccio M. A. Mondelli M. (2010). The first Italian family with tibial muscular dystrophy caused by a novel titin mutation. *J. Neurol.* 257 575–579. 19911250 10.1007/s00415-009-5372-3

[B113] Protonotarios A. Wicks E. Ashworth M. Stephenson E. Guttmann O. Savvatis K. (2019). Prevalence of (18)F-fluorodeoxyglucose positron emission tomography abnormalities in patients with arrhythmogenic right ventricular cardiomyopathy. *Int. J. Cardiol.* 284 99–104. 30409737 10.1016/j.ijcard.2018.10.083

[B114] Puchalski M. D. Williams R. V. Askovich B. Sower C. T. Hor K. H. Su J. T. (2009). Late gadolinium enhancement: precursor to cardiomyopathy in Duchenne muscular dystrophy? *Int. J. Cardiovasc. Imaging* 25 57–63. 18686011 10.1007/s10554-008-9352-y PMC2746925

[B115] Puzzi L. Borin D. Gurha P. Lombardi R. Martinelli V. Weiss M. (2019). Knock down of plakophillin 2 dysregulates adhesion pathway through upregulation of miR200b and alters the mechanical properties in cardiac cells. *Cells* 8:1639. 10.3390/cells8121639 PMC6952926 31847412

[B116] Quarta G. Syrris P. Ashworth M. Jenkins S. Zuborne Alapi K. Morgan J. (2012). Mutations in the Lamin A/C gene mimic arrhythmogenic right ventricular cardiomyopathy. *Eur. Heart J.* 33 1128–1136. 22199124 10.1093/eurheartj/ehr451

[B117] Rampazzo A. Nava A. Malacrida S. Beffagna G. Bauce B. Rossi V. (2002). Mutation in human desmoplakin domain binding to plakoglobin causes a dominant form of arrhythmogenic right ventricular cardiomyopathy. *Am. J. Hum. Genet.* 71 1200–1206. 12373648 10.1086/344208 PMC385098

[B118] Richardson P. McKenna W. Bristow M. Maisch B. Mautner B. O’Connell J. (1996). Report of the 1995 world health organization/international society and federation of cardiology task force on the definition and classification of cardiomyopathies. *Circulation* 93 841–842. 8598070 10.1161/01.cir.93.5.841

[B119] Rizzo S. Lodder E. M. Verkerk A. O. Wolswinkel R. Beekman L. Pilichou K. (2012). Intercalated disc abnormalities, reduced Na(+) current density, and conduction slowing in desmoglein-2 mutant mice prior to cardiomyopathic changes. *Cardiovasc. Res.* 95 409–418. 22764152 10.1093/cvr/cvs219

[B120] Ross S. E. Hemati N. Longo K. A. Bennett C. N. Lucas P. C. Erickson R. L. (2000). Inhibition of adipogenesis by Wnt signaling. *Science* 289 950–953. 10937998 10.1126/science.289.5481.950

[B121] Rybakova I. N. Patel J. R. Ervasti J. M. (2000). The dystrophin complex forms a mechanically strong link between the sarcolemma and costameric actin. *J. Cell Biol.* 150 1209–1214. 10974007 10.1083/jcb.150.5.1209 PMC2175263

[B122] Sato P. Y. Musa H. Coombs W. Guerrero-Serna G. Patino G. A. Taffet S. M. (2009). Loss of plakophilin-2 expression leads to decreased sodium current and slower conduction velocity in cultured cardiac myocytes. *Circ. Res.* 105 523–526. 19661460 10.1161/CIRCRESAHA.109.201418 PMC2742576

[B123] Segura-Rodriguez D. Bermudez-Jimenez F. J. Carriel V. Lopez-Fernandez S. Gonzalez-Molina M. Oyonarte Ramirez J. M. (2019). Myocardial fibrosis in arrhythmogenic cardiomyopathy: a genotype-phenotype correlation study. *Eur. Heart J. Cardiovasc. Imaging* 21 378–386. 10.1093/ehjci/jez277 31702781

[B124] Sen-Chowdhry S. McKenna W. J. (2008). The utility of magnetic resonance imaging in the evaluation of arrhythmogenic right ventricular cardiomyopathy. *Curr. Opin. Cardiol.* 23 38–45. 18281826 10.1097/HCO.0b013e3282f2c96e

[B125] Sen-Chowdhry S. Syrris P. Prasad S. K. Hughes S. E. Merrifield R. Ward D. (2008). Left-dominant arrhythmogenic cardiomyopathy: an under-recognized clinical entity. *J. Am. Coll. Cardiol.* 52 2175–2187. 19095136 10.1016/j.jacc.2008.09.019

[B126] Sen-Chowdhry S. Syrris P. Ward D. Asimaki A. Sevdalis E. McKenna W. J. (2007). Clinical and genetic characterization of families with arrhythmogenic right ventricular dysplasia/cardiomyopathy provides novel insights into patterns of disease expression. *Circulation* 115 1710–1720. 17372169 10.1161/CIRCULATIONAHA.106.660241

[B127] Shang Y. C. Wang S. H. Xiong F. Peng F. N. Liu Z. S. Geng J. (2016). Activation of Wnt3a signaling promotes myogenic differentiation of mesenchymal stem cells in mdx mice. *Acta Pharmacol. Sin.* 37 873–881. 27133298 10.1038/aps.2016.38 PMC4933759

[B128] Sheikh F. Ross R. S. Chen J. (2009). Cell-cell connection to cardiac disease. *Trends Cardiovasc. Med.* 19 182–190. 20211433 10.1016/j.tcm.2009.12.001 PMC3601820

[B129] Silva M. C. Meira Z. M. Gurgel Giannetti J. da Silva M. M. Campos A. F. Barbosa Mde M. (2007). Myocardial delayed enhancement by magnetic resonance imaging in patients with muscular dystrophy. *J. Am. Coll. Cardiol.* 49 1874–1879. 17481447 10.1016/j.jacc.2006.10.078

[B130] Spencer M. J. Montecino-Rodriguez E. Dorshkind K. Tidball J. G. (2001). Helper (CD4(+)) and cytotoxic (CD8(+)) T cells promote the pathology of dystrophin-deficient muscle. *Clin. Immunol.* 98 235–243. 11161980 10.1006/clim.2000.4966

[B131] Spencer M. J. Tidball J. G. (2001). Do immune cells promote the pathology of dystrophin-deficient myopathies? *Neuromuscul. Disord.* 11 556–564. 11525885 10.1016/s0960-8966(01)00198-5

[B132] Swope D. Li J. Radice G. L. (2013). Beyond cell adhesion: the role of armadillo proteins in the heart. *Cell Signal.* 25 93–100. 23022961 10.1016/j.cellsig.2012.09.025 PMC3508382

[B133] Syrris P. Ward D. Evans A. Asimaki A. Gandjbakhch E. Sen-Chowdhry S. (2006). Arrhythmogenic right ventricular dysplasia/cardiomyopathy associated with mutations in the desmosomal gene desmocollin-2. *Am. J. Hum. Genet.* 79 978–984. 17033975 10.1086/509122 PMC1698574

[B134] Tatsumi R. Sankoda Y. Anderson J. E. Sato Y. Mizunoya W. Shimizu N. (2009). Possible implication of satellite cells in regenerative motoneuritogenesis: HGF upregulates neural chemorepellent Sema3A during myogenic differentiation. *Am. J. Physiol. Cell Physiol.* 297 C238–C252. 19515904 10.1152/ajpcell.00161.2009

[B135] Taylor M. Graw S. Sinagra G. Barnes C. Slavov D. Brun F. (2011). Genetic variation in titin in arrhythmogenic right ventricular cardiomyopathy-overlap syndromes. *Circulation* 124 876–885. 21810661 10.1161/CIRCULATIONAHA.110.005405 PMC3167235

[B136] Te Riele A. S. Agullo-Pascual E. James C. A. Leo-Macias A. Cerrone M. Zhang M. (2017). Multilevel analyses of SCN5A mutations in arrhythmogenic right ventricular dysplasia/cardiomyopathy suggest non-canonical mechanisms for disease pathogenesis. *Cardiovasc. Res.* 113 102–111. 28069705 10.1093/cvr/cvw234 PMC5220677

[B137] Thibaud J. L. Monnet A. Bertoldi D. Barthelemy I. Blot S. Carlier P. G. (2007). Characterization of dystrophic muscle in golden retriever muscular dystrophy dogs by nuclear magnetic resonance imaging. *Neuromuscul. Disord.* 17 575–584. 17537632 10.1016/j.nmd.2007.03.013

[B138] Thiene G. Corrado D. Basso C. (2007). Arrhythmogenic right ventricular cardiomyopathy/dysplasia. *Orphanet. J. Rare Dis.* 2:45. 10.1186/1750-1172-2-45 18001465 PMC2222049

[B139] Thiene G. Corrado D. Nava A. Rossi L. Poletti A. Boffa G. M. (1991). Right ventricular cardiomyopathy: is there evidence of an inflammatory aetiology?. *Eur. Heart J.* 12 (Suppl. D), 22–25. 10.1093/eurheartj/12.suppl_d.22 1915454

[B140] Tiso N. Stephan D. A. Nava A. Bagattin A. Devaney J. M. Stanchi F. (2001). Identification of mutations in the cardiac ryanodine receptor gene in families affected with arrhythmogenic right ventricular cardiomyopathy type 2 (ARVD2). *Hum. Mol. Genet.* 10 189–194. 11159936 10.1093/hmg/10.3.189

[B141] Towbin J. A. McKenna W. J. Abrams D. J. Ackerman M. J. Calkins H. Darrieux F. C. C. (2019). 2019 HRS expert consensus statement on evaluation, risk stratification, and management of arrhythmogenic cardiomyopathy: Executive summary. *Heart Rhythm* 16 e373–e407. 31676023 10.1016/j.hrthm.2019.09.019

[B142] Turkowski K. L. Tester D. J. Bos J. M. Haugaa K. H. Ackerman M. J. (2017). Whole exome sequencing with genomic triangulation implicates CDH2-encoded N-cadherin as a novel pathogenic substrate for arrhythmogenic cardiomyopathy. *Congenit. Heart Dis.* 12 226–235. 28326674 10.1111/chd.12462

[B143] Uezumi A. Fukada S. Yamamoto N. Takeda S. Tsuchida K. (2010). Mesenchymal progenitors distinct from satellite cells contribute to ectopic fat cell formation in skeletal muscle. *Nat. Cell Biol.* 12 143–152. 20081842 10.1038/ncb2014

[B144] Uezumi A. Ito T. Morikawa D. Shimizu N. Yoneda T. Segawa M. (2011). Fibrosis and adipogenesis originate from a common mesenchymal progenitor in skeletal muscle. *J. Cell Sci.* 124(Pt 21), 3654–3664. 22045730 10.1242/jcs.086629

[B145] van der Heijden J. F. Hassink R. J. (2013). The phospholamban p.Arg14del founder mutation in Dutch patients with arrhythmogenic cardiomyopathy. *Neth Heart J.* 21 284–285. 23595706 10.1007/s12471-013-0413-z PMC3661881

[B146] van der Zwaag P. A. van Rijsingen I. A. Asimaki A. Jongbloed J. D. van Veldhuisen D. J. Wiesfeld A. C. (2012). Phospholamban R14del mutation in patients diagnosed with dilated cardiomyopathy or arrhythmogenic right ventricular cardiomyopathy: evidence supporting the concept of arrhythmogenic cardiomyopathy. *Eur. J. Heart Fail.* 14 1199–1207. 22820313 10.1093/eurjhf/hfs119 PMC3475434

[B147] van der Zwaag P. A. van Rijsingen I. A. de Ruiter R. Nannenberg E. A. Groeneweg J. A. Post J. G. (2013). Recurrent and founder mutations in the Netherlands-phospholamban p.Arg14del mutation causes arrhythmogenic cardiomyopathy. *Neth. Heart J.* 21 286–293. 23568436 10.1007/s12471-013-0401-3 PMC3661879

[B148] van Hengel J. Calore M. Bauce B. Dazzo E. Mazzotti E. De Bortoli M. (2013). Mutations in the area composita protein alphaT-catenin are associated with arrhythmogenic right ventricular cardiomyopathy. *Eur. Heart J.* 34 201–210. 23136403 10.1093/eurheartj/ehs373

[B149] van Opbergen C. J. M. Noorman M. Pfenniger A. Copier J. S. Vermij S. H. Li Z. (2019). Plakophilin-2 haploinsufficiency causes calcium handling deficits and modulates the cardiac response towards stress. *Int. J. Mol. Sci.* 20:4076. 10.3390/ijms20174076 PMC6747156 31438494

[B150] van Spaendonck-Zwarts K. Y. van Hessem L. Jongbloed J. D. de Walle H. E. Capetanaki Y. van der Kooi A. J. (2011). Desmin-related myopathy. *Clin. Genet.* 80 354–366. 20718792 10.1111/j.1399-0004.2010.01512.x

[B151] van Tintelen J. P. Van Gelder I. C. Asimaki A. Suurmeijer A. J. Wiesfeld A. C. Jongbloed J. D. (2009). Severe cardiac phenotype with right ventricular predominance in a large cohort of patients with a single missense mutation in the DES gene. *Heart Rhythm* 6 1574–1583. 19879535 10.1016/j.hrthm.2009.07.041

[B152] Verma M. Asakura Y. Murakonda B. S. R. Pengo T. Latroche C. Chazaud B. (2018). Muscle satellite cell cross-talk with a vascular niche maintains quiescence via VEGF and notch signaling. *Cell Stem Cell.* 23 530–43e9. 30290177 10.1016/j.stem.2018.09.007 PMC6178221

[B153] Vetrone S. A. Montecino-Rodriguez E. Kudryashova E. Kramerova I. Hoffman E. P. Liu S. D. (2009). Osteopontin promotes fibrosis in dystrophic mouse muscle by modulating immune cell subsets and intramuscular TGF-beta. *J. Clin. Invest.* 119 1583–1594. 19451692 10.1172/JCI37662 PMC2689112

[B154] Villalta S. A. Rosenberg A. S. Bluestone J. A. (2015). The immune system in Duchenne muscular dystrophy: Friend or foe. *Rare Dis.* 3:e1010966. 10.1080/21675511.2015.1010966 PMC4588155 26481612

[B155] Vita G. L. Polito F. Oteri R. Arrigo R. Ciranni A. M. Musumeci O. (2018). Hippo signaling pathway is altered in Duchenne muscular dystrophy. *PLoS One* 13:e0205514. 10.1371/journal.pone.0205514 30304034 PMC6179272

[B156] Wehling-Henricks M. Lee J. J. Tidball J. G. (2004). Prednisolone decreases cellular adhesion molecules required for inflammatory cell infiltration in dystrophin-deficient skeletal muscle. *Neuromuscul. Disord.* 14 483–490. 15336689 10.1016/j.nmd.2004.04.008

[B157] Wei B. Dui W. Liu D. Xing Y. Yuan Z. Ji G. (2013). MST1, a key player, in enhancing fast skeletal muscle atrophy. *BMC Biol.* 11:12. 10.1186/1741-7007-11-12 23374633 PMC3606410

[B158] Whitehead N. P. Yeung E. W. Allen D. G. (2006). Muscle damage in mdx (dystrophic) mice: role of calcium and reactive oxygen species. *Clin. Exp. Pharmacol. Physiol.* 33 657–662. 16789936 10.1111/j.1440-1681.2006.04394.x

[B159] Williams I. A. Allen D. G. (2007). Intracellular calcium handling in ventricular myocytes from mdx mice. *Am. J. Phys. Heart Circ. Physiol.* 292 H846–H855. 10.1152/ajpheart.00688.2006 17012353

[B160] Yasuda S. Townsend D. Michele D. E. Favre E. G. Day S. M. Metzger J. M. (2005). Dystrophic heart failure blocked by membrane sealant poloxamer. *Nature* 436 1025–1029. 16025101 10.1038/nature03844

[B161] Yeung E. W. Whitehead N. P. Suchyna T. M. Gottlieb P. A. Sachs F. Allen D. G. (2005). Effects of stretch-activated channel blockers on [Ca2+]i and muscle damage in the mdx mouse. *J. Physiol.* 562(Pt 2), 367–380. 15528244 10.1113/jphysiol.2004.075275 PMC1665499

[B162] Yin L. Xie Z. Y. Xu H. Y. Zheng S. S. Wang Z. X. Xiao J. X. (2019). T2 mapping and fat quantification of thigh muscles in children with duchenne muscular dystrophy. *Curr. Med. Sci.* 39 138–145. 30868504 10.1007/s11596-019-2012-8

[B163] Zhou Q. Li L. Zhao B. Guan K. L. (2015). The hippo pathway in heart development, regeneration, and diseases. *Circ. Res.* 116 1431–1447. 25858067 10.1161/CIRCRESAHA.116.303311 PMC4394208

